# The Ottawa Citizen Engagement and Action Model (OCEAM): A Citizen engagement Strategy Operationalized Through The Participatory Research in Ottawa, Management and Point-of-care of Tobacco (PROMPT) Study: A Community Based Participatory Action Research Project in Inner City Ottawa

**DOI:** 10.1186/s40900-016-0034-y

**Published:** 2016-05-21

**Authors:** Smita Pakhale, Tina Kaur, Kelly Florence, Tiffany Rose, Robert Boyd, Joanne Haddad, Donna Pettey, Wendy Muckle, Mark Tyndall

**Affiliations:** 1 The Ottawa Hospital, University of Ottawa, Ottawa, Canada; 2 The Ottawa Hospital Research Institute (OHRI), University of Ottawa, Ottawa, Canada; 3 University of Ottawa, Ottawa, Canada; 4 Community research peers, Oasis, Sandy Hill Community Health Centre, Ottawa, Canada; 5 Oasis, Sandy Hill Community Health Centre, Ottawa, Canada; 6 Canadian Mental Health Association, Ottawa Branch, Ottawa, Canada; 7 Ottawa Inner City Health Inc., Ottawa, Canada; 8 British Columbia CDC, University of British Columbia, Vancouver, BC Canada; 9 University of British Columbia, Vancouver, BC Canada

**Keywords:** Knowledge Translation, Nicotine Replacement Therapy, City Population, Smoking Cessation Program, Tobacco Dependence

## Abstract

**Plain language summary:**

The PROMPT study is a community-based research project designed to understand the factors which affect smoking as well as ways to manage, reduce and quit smoking among people who use drugs in Ottawa. There is strong medical evidence that smoking tobacco is related to more than two dozen diseases and conditions. Smoking tobacco remains the leading cause of preventable death and has negative health impacts on people of all ages. Although Ottawa has one of the lowest smoking rates in Ontario (12 %), major differences exist, with approximately a 96 % smoking rate among those who use drugs in the city of Ottawa. To address this inequity, we recruited and trained four community research peers who were representative of the study target population (ex- or currently homeless, insecurely housed or multi-drug users). We designed the ten-step Ottawa Citizen Engagement and Action Model (OCEAM) for the PROMPT study. In this paper we have described this process in a step-by-step fashion, as used in the PROMPT study. The eighty PROMPT participants are being followed for six months and are being provided with free and off-label Nicotine Replacement Therapy (NRT).

**Abstract:**

**Objectives** The PROMPT study, Participatory Research in Ottawa, Management and Point-of-care of Tobacco, is a prospective cohort study which utilizes community-based participation and social network-based approaches to address tobacco dependence in inner city Ottawa. The project was designed to: facilitate retention of participants; to understand the barriers and facilitators of smoking; optimize ways to manage, reduce, and quit tobacco use among people who use drugs in Ottawa, Canada. The purpose of this paper is to describe the processes utilized in citizen or patient engagement in academic research, through our tobacco dependence management project in the inner city population in Ottawa, Canada.

**Background** Tobacco smoking is inequitably distributed in Canada with rates at 12 % in Ottawa, as compared to 18 % in rest of Canada. However, the PROUD Study (Participatory Research in Ottawa: Understanding Drugs) demonstrated that 96 % of the inner city population, of Ottawa currently smoke tobacco. This distinct inequity in tobacco use translates into inequitable distribution of health outcomes, such morbidity and mortality in this population. Consequently, a community-based participatory, peer-led research project was conducted in the inner city population of Ottawa.

**Methods** We recruited and trained four community research peers who were representative of the study target population. We conceived, designed and operationalized the ten-step Ottawa Citizen Engagement and Action Model (OCEAM) for the PROMPT study. The peers have co-led all aspects of the project from conceptualizing the study question to participating in knowledge translation. Each step of the project had defined objectives and outcome measures.

**Discussion** The involvement of peers in recruitment ensured representation of tobacco and drug users—individuals truly representative of the intended target population. Peer, participant engagement and trust was established from the conception of the project. For historical and self-evident reasons, trust and engagement is rarely found in this population. Peers successfully participated in all ten steps of the Citizen Engagement and Action model. The PROMPT study utilized the CBPR (Community Based Participatory research) approach to encourage engagement and build trust in a difficult to reach and hard to treat, inner city population. The ten-step OCEAM model was conceived, designed and operationalized and the PROMPT study will continue to follow the eighty PROMPT participants for six months to understand the optimal ways to manage, reduce, and quit smoking within an inner city population.

## Background

The complexities of the health problems faced by inner city, slum, or socially segregated populations are poorly suited for traditional, “outside expert-driven research and intervention approaches” [1]. Alternate approaches are urgently needed to address the various health issues disproportionally affecting these populations. Community-Based Participatory Research (CBPR) is an alternative approach in which researchers and community stakeholders form equitable partnerships to tackle issues related to community health improvement and knowledge production [2, 3]. CBPR is based on two primary assumptions for improving health outcomes and reducing disparities. Firstly, interventions can be strengthened if they benefit from community insight and incorporate community values. Secondly, there is added value to participation itself as it enhances health [4]. Participatory Action Research (PAR) is well-rooted in the early 19th century when a well-known American philosopher, John Dewey eloquently described the association between knowledge and action [5, 6]. John Dewey’s philosophy was put into action by one of his famous students, Dr. B.R. Ambedkar, during his work with issues surrounding casteism and exploration of discrimination against the lowest caste groups in India. In particular, Ambedkar’s actions were based on fact finding and critical reflections [7]. Kurt Lewin, a social psychologist, and John Collier, a social worker and anthropologist, also expanded on these ideas of participatory research in 1940s in the United States [8]. Greater, more meaningful participation in research is being called for by peer-led drug advocacy action groups in Canada, “Nothing For Us, Without Us” [9]. Although there have been efforts to more meaningfully engage target communities in research, the affected community is still rarely involved in every step of the development, design, and dissemination of research projects [10].

The goal of participation in community development literature is to reduce dependency on health professionals, to ensure cultural and local sensitivity, to facilitate sustainability and to enhance productivity of programs. [11, 12] The health impacts of participation, however, remain largely elusive. There is a body of work that problematizes community-based research in terms of empowerment and the tyranny of participation [4]. However, research on the effectiveness of participatory strategies within the empowerment literature has demonstrated the effects of participation and empowerment and the positive influence it has on improving health [4].

The literature shows strong evidence that community participation contributes to program improvement through greater efficiency, sustainability, and more equitable distribution of services [2, 3, 13–15]. Only a few published studies have tested designs to valídate the hypothesis that community participation provides additional health benefits. In Eng, Briscoe, and Cunningham’s (1990) landmark study in Indonesia and Togo, villages where water was installed with active community participation found that 25 to 30 % more children were immunized, than in villages where there was no active community participation [16]. This example provides insight into the unintended health benefits as a result of community participation. There are various challenges of studying community participation within community settings, where local context matters, dynamic processes are assumed, and participatory feedback is crucial to have an effective intervention [4].

Ideally, CBPR in public health is a partnership approach to research that equitably involves the community at large, for example, community members, organizational representatives, and researchers are involved in all aspects of the research process, in which all partners contribute their expertise,share decision making and responsibilities [17, 18]. The aim of CBPR is to increase knowledge and understanding of a given phenomenon, integrate the knowledge gained with interventions and influence policy change to improve the health of communities at large. Within the context of CBPR, community is defined as a unit of identity [17, 18]. Based on an extensive review of the literature [18], a list of eight principles or characteristics of CBPR have been identified: These include: i) recognizing community as a unit of identity; ii) building on strengths and resources within the community; iii) facilitating collaborative partnerships in all phases of the research; iv) integrating knowledge and action for mutual benefit of all partners; v) promoting a co-learning and empowering process that attends to social inequalities; vi) involving a cyclical and iterative process; vii) addressing health from both positive and ecological perspectives and viii) disseminating findings and knowledge gained to all partners.

Researchers today are increasingly turning to CBPR approaches as a framework in which to conduct research. There is a growing recognition that “traditional” research approaches have failed to solve complex health disparities [4]. Many research designs fail to incorporate multi-level explanations of health and the researchers themselves do not understand many of the social and economic complexities motivating individual and community behaviours. Community members themselves, weary of being “guinea pigs”, are increasingly demanding that research address their locally identified needs. Traditional researchers often complain about challenges in trying to recruit “research subjects”. These challenges are often a result of community members feeling that researchers have used them and taken findings away for the researchers benefit (e.g., scholarly papers) but the community is left with no direct benefit [4, 17].

Through the concerted efforts of practitioners and policy makers, the prevalence of tobacco-use has been reduced to 18 % in Canada over the past several decades. Although Ottawa has one of the lowest smoking rates in Ontario at 12 % [19], major disparities exist within the population as disproportionately higher rates of smoking were observed among drug users and individuals with addictions. In the recent PROUD study of Ottawa inner city residents who used multiple drugs, 96 % had smoked cigarettes in the past year [20]. The common assumption is that people who use drugs do not want to quit smoking; however, various studies have documented that approximately 44-80 % of drug users are interested in quitting [21, 22]. According to the literature, the majority of smokers (72 %) reported that they had tried to quit smoking previously, 69 % expressed interest in participating in a group smoking cessation program, while 82 % indicated interest in receiving a prescription for a nicotine replacement medication. A majority of smokers considering cessation (56 %) reported that they were interested in both group intervention and nicotine replacement [21, 22]. Therefore, the motivation to quit smoking exists within the community, however there is a lack of comprehensive programs designed to cater to marginalized individuals in Ottawa. There is a need for an adapted, community based smoking cessation program which will engage the community, empower members, provide them with support to quit or reduce their tobacco use and may further encourage other healthy habits (safer drug use, decreased use etc.). Consequently, we conducted a community-based participatory, peer-led action research project in the inner city population of Ottawa at a low-threshold, non-judgmental, safe space located on Murray Street in the ByWard Market area of Ottawa. The purpose of this paper is to describe the processes utilized in citizen or patient engagement in academic research, through our tobacco dependence management project in the inner city population in Ottawa, Canada.

## Methods

The Participatory Research in Ottawa: Management and Point-of-care of Tobacco (PROMPT) research study is a Prospective Cohort Study through which we aim to learn optimal ways to disseminate evidence-based tobacco dependence management in the hardest-to-reach inner city population. The PROMPT study employs Community-Based Participatory Action Research (CBPAR) method integrated within a Social Network approach. Health care interventions can be most efficiently diffused by exploiting the intrinsic properties of human social networks [23]. Information is transmitted and distributed through friends and social networks within communities. Insecurely housed, homeless populations of inner cities in North America, or slums and segregated populations elsewhere, have tight-knit social networks with unique characteristics where consistent information exchanges occur throughout the network. We exploited these social networks of peers and participants for recruitment and retention in the PROMPT study. The overall aim of this proposed approach is to understand the barriers and facilitators of smoking as well as to assess the lung health of participants using novel techniques, while incorporating the CBPR method.

PROMPT was exclusively conducted at a Community Research Centre, located in downtown Ottawa in the neighborhood of our study target population — a safe, low-threshold, and a non-judgmental space for the community peers and participants. It was critical to integrate and involve those who use drugs in Ottawa to better understand the structural, environmental and cultural norms which lead to high rates of tobacco use within this community.

### The Ottawa Citizen Engagement and Action Model (OCEAM)

We conceived, designed and operationalized the ten-step Ottawa Citizen Engagement and Action Model in the PROMPT study (Table 1). From the inception of the project, we actively engaged community peers, truly representative of the study target population. By ‘citizen engagement’, we mean the same as in ‘patient’ or ‘citizen’ engagement envisioned in Canada, in the CIHR-SPOR (Strategic Patient Oriented Research) [24, 25]. The ten steps of peer or public or citizen or patient engagement and action are as follows:

**Table 1:** The ten steps of the Ottawa Citizen Engagement and Action Model (OCEAM)

Ten Steps	Outcome Measures	Threshold of Success	Timeframe
1. Formulating a relevant study question	A Study question relevant to the community; and Recruitment and retention of engaged peers	Successful selection of a relevant study question and recruitment of 4-6 engaged peers, throughout the project	3–6 months (more if no pre-existing community ties)
2. Designing study method	A study design	Agreed upon study method by peers and researchers that is sufficiently rigorous; Successful implementation of study method with peers	3 months
3. Designing study questionnaires and Case Report Forms (CRFs)	Study questionnaires and CRFs	Successful design and selection of questionnaires and CRF that peers are satisfied with	3 months
4. Participating in recruitment	Recruitment of sample	Successful and efficient (within the decided accrual time) recruitment of at least 80 % of envisioned sample size	3 months of the accrual time decided for the project
5. Participating in consenting	Participants written Consent	Consenting of at least 80 % potential participants	3 months of the accrual time decided for the project
6. Participating in administering study questionnaires	Completed questionnaires	Less than 30 % missing data per entire questionnaire and CRFs	3 months of the accrual time decided for the project
7. Participating in study related testing e.g.;handheld spirometry and oscillometry	Testing completed	Successful implementation of acceptable quality study tests with less than 30 % missing data	3 months of the accrual time decided for the project
8. Participating in follow-ups	Participant retention at study completion	i) Peer participation in retention with follow-up rates of at least 60 % ii) Peer (at least 1–2) and participant (at least 6–10) attendance at retention related activities such as Life-Skills Workshop of PROMPT study	3 months of the follow-up time decided for the project
9. Participating in data entry, data analysis and interpretation	Peer participation in data entry, data analysis and interpretation	At least 50 % peers participating in data entry, and 25 % peers participating in data analysis and interpretation of study results	3–6 months after completion of follow-ups
10. Participating in ongoing community knowledge translation	Continuous knowledge translation through: i) Peer training (six sessions), ii) Regular project meetings with peers (at least weekly), iii) Peer-led community knowledge forums (quarterly)	i) At least 80 % peers attending all 6 training sessions ii) At least 50 % of peers attending weekly meetings iii) At least 50 % of peers participating in quarterly community knowledge forums	Ongoing throughout the life of the project

#### Formulating a relevant study question

##### Building the community-based participatory research team

An initial meeting was held in April 2014 with academic and community members. The purpose of this meeting was to share the academic researchers’ vision and community perspective. Tobacco was felt to be an important issue by the community after the PROUD study demonstrated that 96 % of the population smokes tobacco. Hence, peer involvement in conceptualizing and designing the study question was the starting point of the project and pivotal in strengthening our bonds between the academic team and community peer researchers. Similar to PROUD, we partnered with community grassroot organizations, created by and for the people who use multiple drugs. Through this partnership, we invited community peers from the study target population to participate in the project. Community members were eligible to apply for the peer researcher position if they belonged to the target study population (i.e., with current or past drug use, ex- or current tobacco smoke and who are/were homeless or insecurely housed). Peers were interviewed by two people (a community organization member and an academic physician) at the community research centre. We interviewed nine aspiring peers and selected four of them. Selection was based upon genuineness of their membership to the study target population, their experience with the community, their individual social networks within the study target population, as well as their commitment towards community capacity building. Commitment was determined by their potential ability to commit to the project long-term and their engagement within the community. Of the four selected peers, two had experience with recruiting for the PROUD project, one had worked with knowledge dissemination of PROUD and one was new to community research. Selected peer researchers were offered an honorarium of $15 per hour (25 % above the minimum wage in Ontario) for every hour spent working on the project for interviews, training and other project related meetings. Successful recruitment and retention of at least four engaged peers was an expected outcome.

#### Designing the study method

Biweekly meetings with academic and peer researchers were organized. In the initial phase of the study the meetings served to develop a shared vision for the project. The meetings also ensured concerns were addressed and that there was shared decision-making and collaboration throughout all stages of the study. The study design as well as survey development were addressed further once an agreement on the shared vision was achieved. PROMPT, designed to be a prospective cohort study, envisioned to recruit 80 participants and follow them for six months. The participants were to receive one-on-one counseling from a smoking cessation expert nurse and free and off-label nicotine replacement therapy. Other main topics addressed at this stage included peer researcher training, distribution or redistribution of tasks, study method details such as participant eligibility, different recruitment strategies, effectively utilizing peers’ social networks for recruitment and retention, consent and data collection. Success of recruitment and retention was heavily based upon peer engagement, peers’ social networks and community outreach through neighbourhood healthcare organizations. Participation and buy-in of the research peers on study design, method, and operationalization was key to the project. Thus, though the study design remained the same, our approach towards implementation was modified through peer participation.

#### Designing study questionnaires and Case Report Forms (CRFs)

The initial version for the PROMPT project baseline questionnaire was drafted by the academic researchers. The baseline questionnaire included demographic information, smoking history, drug use history, lung health related issues and clinical history relevant to tobacco smoking. After a series of meetings, lively discussions and debates, questionnaires were refined with the help of the peer researchers and their lived experiences. Suggestions and changes were made to language and the structure of survey questions. Culturally appropriate language editing proved to be crucial, for example, a commonly used item on most respiratory questionnaires reads ‘Feel short of breath when walking on level ground’ was deemed inappropriately worded by the research peers. Because, ‘on level ground’ has a different interpretation on the streets; during one of our tool development meeting, the peers explained that ‘O*n level ground for us means not being under the influence of drugs or alcohol, but being level headed’!* Thus, the wording was appropriately amended to address such concerns. With peers, the social network questionnaire was designed and finalized with the research team, in order to formally study social networks in our target population. The social network questionnaire captured information on 13 dimensions, including names of friends or relatives who visit the respondent’s living space, names of those friends or relatives whom the respondent visits or goes to pray with (at a church, mosque or temple), from whom the respondent would borrow or lend money, from whom the respondent would borrow or to whom the respondent would lend material goods to (food, cigarettes), and from whom the respondent receives or gives advice to.

Finally, we came to a consensus to administer the following six questionnaires to participants at baseline: 1) demographic, detailed smoking history, drug use questionnaire and the social network questionnaire, 2) Fagerstorm Test for Nicotine Dependence (FTND) [26], 3) the BOLD core questionnaire used in the CanCOLD study, which aims to evaluate respiratory symptoms (cough, phlegm, whistling/wheezing, shortness of breath) [27, 28], 4) Chronic Obstructive Pulmonary Disease (COPD) Assessment Test (CAT), an open-access disease-specific questionnaire [29], 5) EQ-5D, a well-validated five item open access questionnaire which measures generic quality of life [30], and 6) Patient Health Questionnaire (PHQ-8), an eight-item open-access questionnaire which is used to establish provisional depressive disorder diagnoses as well as grade depressive symptom severity [31]. The goal of this step was to select study questionnaires and CRFs in consensus with the peer researchers and ensure there was peer agreement.

##### Peer training

The peer researchers underwent rigorous training on different strategies for recruitment, consenting, administration of baseline surveys and social network items as well as administration of spirometry and osillometry. The spirometry training followed the CanCOLD study training guidelines [32]. The peer training was led by a Respirologist trained in pulmonary function testing (SP) and the training was adapted to the their level of knowledge of lung function. All peers underwent six group sessions and a one-on-one training session. The group sessions included didactic presentations, discussions, role playing and practice. The one-on-one session was focused on the practice of performing the spirometry test and questionnaire administration. In addition to lung health knowledge and interviewing skills, the peer training focused on issues related to consent such as; confidentiality, autonomy, privacy, the Tri-Council Policy Statement 2, verbal and non-verbal communication; diversity of the study participants; general ethical concerns of research in marginalized populations; and health disparity literature updates. The peers underwent pre- and post-training workshop surveys and demonstrated that their knowledge and skills were significantly improved once the training was completed (Unpublished).

#### Participating in recruitment

Peer participation in recruitment was the key in order to enroll genuine members of the target population. Social network based recruitment was undertaken through the well-established social networks of peers, local healthcare agencies, drop-in centres, shelters, Ottawa Inner City Health Inc., and Ottawa Public Health outreach programs. Participant recruitment was mainly limited to the Ottawa ByWard Market downtown area. Recruitment and enrollment started in March 2015 and eighty participants were enrolled in the study by mid-August. Participants were eligible if they were 16 years or older, using multiple drugs other than marijuana and alcohol, currently smoked tobacco and had been living in Ottawa for at least 3 months. Participants were excluded if they were currently enrolled or had participated in any other smoking cessation program in the last 30 days. Participation was entirely voluntary. Motivation and willingness to quit as well as accessibility to come to follow-ups was evaluated and determined by peers during recruitment. The criteria for determining motivation to quit smoking and motivation to follow up were left to the discretion of the peers. When participants were known to peers this was assessed rapidly. When participants were not known to the peers a more lengthy discussion took place to obtain a general impression of their commitment before their enrollment in the project. Success at this step was evaluated by efficient enrollment of the envisioned sample size.

#### Participating in consent

Consent was obtained by peers at the time of initial intake. The participants were asked to consent to completing the survey, the lung function tests and the data linkages. Through the data linkages, the PROMPT cohort would be followed prospectively for one year through the Institute of Clinical and Evaluative Sciences (ICES) which collects ongoing publicly funded health care data for Ontario residents. The research team will obtain linkable data sets to understand smoking associated health care utilization among participants in the PROMPT cohort. Participants were able to opt out of either the lung function tests i.e., spirometry and oscillometry or the data linkages through ICES. Recruitment and the initial intake were undertaken on the same day when possible to minimize missed appointments. Active involvement of peers in the recruitment and consenting process reinforced engagement of participants in the project.

#### Participating in administering study questionnaires

Baseline surveys, testing and follow-up visits were conducted at the community research centre. The initial intake was led by peer researchers and included the iPad-directed baseline survey and social network items as well as the baseline lung function testing. The data were saved to a secure database from the iPad. Consistent with other cohort research projects, a cash honorarium of $20.00 was offered to participants after completing the baseline survey. This was provided even if participants opted out of lung function testing or data linkages. Participants were informed that they could skip any of the questions if they were uncomfortable answering them. A peer-led approach to questionnaire administration was consciously adopted in order to avoid the social desirability bias [33].

In addition, participants were enrolled in the Smoking Treatment for Ontario Patients (STOP) led by the Centre for Addiction and Mental Health (CAMH) in order to offer free and off-label Nicotine Replacement Therapy (NRT). After consenting to the PROMPT study procedures, all participants met with a smoking cessation nurse from the Ottawa chapter of Canadian Mental Health Association (CMHA). The CMHA nurse, whose services were specifically hired for PROMPT study, was available onsite twice a week to offer one-on-one counseling and individualized NRT, available through the STOP program. The participants could meet the nurse as frequently as requested by the participant or as deemed clinically necessary by the nurse. Expired CO was also measured during these visits with a Bedfont Micro Smokerlyzer Carbon Monoxide (CO) monitor (Bedfont Technical Instruments Ltd, Sittingbourne, Kent, United Kingdom) for biochemical confirmation of the self-reported quitting. The step will be considered successful if there is less than 30 % missing data on the questionnaires.

#### Participating in study related testing e.g. handheld spirometry and oscillometry

Peers administered lung function testing which included point of care hand-held spirometry and oscillometry. These were completed before and after administration of 200ug of salbutamol with an aero chamber. Participants with abnormal spirometry and/or oscillometry were encouraged to follow-up with their primary healthcare provider and referred to the Ottawa Hospital for further investigations when appropriate. The step will be considered successful if there is less than 30 % missing data on study measurements.

#### Participating in follow-ups and retention

In addition to the follow-up with the smoking cessation nurse, all participants were encouraged to attend monthly follow-up visits at the project site and received $25 per monthly visit. The short monthly follow up survey was administered either by the project manager or peers as per availability. Participants also dropped in at the ‘French Toast Friday’ breakfast club, hosted regularly at the community research centre in collaboration with our partner community organizations. Close social network of peers encouraged participants’ engagement in the project and thus promoted follow-ups. We discussed challenges (e.g., when a participant was under influence of alcohol or drugs at the follow-up appointment) and opportunities (e.g., contacts of participants at the organization with harm reduction activities in the town) to improve follow-up rates at our regular and ad-hoc peer-meetings. The goal is to achieve at least a 60 % follow-up rate at the end of the six months.

##### Peer-led weekly life skills workshops

To attempt to further engage study participants, weekly, peer-led life-skills workshops are organized and conducted. The workshops are voluntary and all study participants are invited to attend. Some of the organized workshops include: financial literacy, banking, cooking, hepatitis C education, arts, and pet care. These are peer-designed and peer-led workshops and occasionally involve the assistance of volunteers from the general community. At the end of the project, participant satisfaction, knowledge, skills, and self-efficacy data will be collected. Currently, the life-skills workshops are being attended by approximately 6–10 participants and 1–2 peers.

#### Participating in data entry, data analysis and interpretation

Peers were trained in data-entry at the beginning of the project. Ongoing peer training involving a cyclical and iterative process is being used to train peers on data-analysis and interpretation. Peer involvement in data analysis and interpretation was deemed important in order to derive meaningful and relevant conclusions from the data. One of our peer during a meeting stated that, “*We are the end users of the results and hence, we must be involved in the analysis and interpretation so that the results and outcomes are relevant to us.”* Currently, two peers (50 %) are participating in data entry, data analysis and data interpretation.

#### Participating in ongoing community knowledge translation

Knowledge translation is an ongoing aspect of the study. This continuous knowledge translation is achieved through peer training (six sessions), regular project meetings with peers (at least weekly), participant engagement and peer-led community knowledge forums (quarterly).

##### Integrated, ongoing knowledge translation

Key knowledge translation and community capacity building activities are: ongoing peer training, in-service training, debriefing after recruitment, consenting or administration of questionnaires and weekly or biweekly meetings to share our experiences with the progress of the project.

##### Community Knowledge Forums

We are organizing quarterly, peer-designed, peer-led ‘Community Knowledge Forums’ where all stakeholders, partners, funders, peers and participants partake in a lively discussion. To date, three such quarterly forums have been conducted with excellent response and enthusiasm from the peers and participants (May and Sept 2015; and Jan. 2016). We involved and invited community partners and stakeholders to the forums, including staff and key members from neighbourhood community health centres, neighbourhood drop-in centres, shelters, local grassroot organizations, Ottawa Public Health, and Ottawa Inner City Health Inc. During the lively discussion amongst participants and audience members at our first community knowledge forum, a Ottawa Public Health nurse, Ms.E. stated that, “*I am always conflicted as to when is the best time to discuss ‘tobacco issues’ with my clients when they have so many other things going on”* and one of the panelist, a PROMPT project participant, DB, spontaneously said, that ‘*Anytime is a good time!*’. And he added, that, *“We all are fed up with smoking tobacco because of the day-to-day challenges, but it is very hard to quit. Any help is always welcome!”* During our second community knowledge forum, one of our panelists, a PROMPT project participant, JB confessed that, *“My chronic back pain is so much better now that I am smoking only 2-3 cigarettes, I cannot wait till I get over with this.”*

##### Posters, news-items and manuscript writing

The principal investigator, research coordinator and peer researchers along with community partners have formed a writing committee to create materials for the project in print. To date our writing group has created hand-outs for the forums, news-items for local media, submitted two conference abstracts and are currently working on two manuscripts.

##### Community capacity building activities and focus groups at the community research centre

To build community capacity and maximize the use of the community research centre, we are using the space to conduct focus groups on lung health and health literacy. In an attempt to further understand lung health of the study population, a 2-hour peer-led focus group was organized with 8–10 participants from our study population to discuss lung health and research priorities. The participants for the focus group were selected from the PROMPT study population based on their lung function (participants fulfilling the diagnostic criteria for Chronic Obstructive Pulmonary Disease (COPD)). Participation in the focus group was voluntary and consent was obtained at the time of participation. The two peers who led the focus group and the eight participants were compensated with a cash honorarium of $60.

Another focus group was conducted with participants from the same target population as our study, specifically focusing on injection drug use, in Feb 2015 to understand “Harm Reduction in Ontario’s Federal Prisons (Ottawa)”. This focus group was led by the department of Criminology, at Toronto’s Ryerson University, in collaboration with peers from the Prisoners with HIV/AIDS Support Action Network (PASAN), along with the Canadian HIV/AIDS Legal Network and the Native Youth Sexual Health Network. All five participants received $30 honorarium for their time, food and refreshments at the session, and public transit tickets.

On all Friday mornings, in collaboration with local community organizations, we host a ‘French Toast Friday’ breakfast, where all are welcome from the community for a warm breakfast, interaction with familiar faces and also information and educational resources. An Ottawa Public Health nurse is usually in attendance at these breakfast session to deliver some informal health awareness, educational sessions or conduct workshops. Also, a POPP Party (Peer Overdose Prevention Program) was conducted in Feb 2015 to educate opiate users and to promote use of Naloxone Kits created by the Ottawa Public Health office. Between the POPP Party and the ‘French Toast Friday’ breakfast sessions, thus far, fifteen Naloxone kits have been distributed to community members at the research centre within one year.

Peers and participants organized a Thanksgiving dinner and a Christmas dinner in October and December 2015, respectively. On both these occasions, the project participants and peers did the preparation, grocery shopping and cooking. A participant involved in cooking the dinner on Thanksgiving said that, *“It is so nice for me to be here. I am here since 8 AM. If not for here, I would be looking for drugs and what not. I feel so great!”* A participant who did cooking for the dinner in December said that, *“I have been getting night mares about this day since last week and I am preparing myself for it. For all last week, I did not do any crack or any drugs. I had to be ready for today man!”*

## Discussion

The PROMPT study has operationalized a program designed to respond to health inequity in tobacco use, by utilizing community-based participation. Despite almost a 100 % tobacco use rate, we still lack knowledge of COPD prevalence or the effects of multi-drug use on COPD, primarily because, the most marginalized populations have not been included in the major national cohort studies [27].

The ten-step Ottawa Citizen Engagement and Action Model (OCEAM) is being successfully conceived, designed and operationalized in the PROMPT prospective cohort study. Each step has defined objectives and outcome measures. The PROMPT study is designed to support inner city residents, in order to quit or reduce smoking tobacco, encourage participation within their community, and to further build their individual capacity.

In 1978, the World Health Organization’s Alma-Ata Declaration first articulated the goals of community participation and equity, with subsequent extension to empowerment in the Ottawa Charter and Jakarta health promotion declarations. Much research has accrued on the interconnectedness of psychological empowerment, level of participation and a sense of community (i.e., people’s identification and bonding with their community network, social networks or place of residence) [34]. In the PROMPT study, CBPAR embedded in the social network-based approach was adopted as a framework to conduct our peer led tobacco dependence management program. This approach allowed for recruitment of this difficult-to-reach and difficult-to-treat population. Building upon the PROUD study procedures [20], we operationalized a peer-led prospective cohort study design. The collaboration with the inner city population of Ottawa was facilitated by our partnership with the neighbourhood health care agencies and grassroots organizations serving the same target population. This was a great opportunity to strengthen relationships between the academic community and the inner city population and to further build community capacity and trust.

Effective interventions ensure supportive environments are created by engaging key stakeholders and community leaders. Supportive environments are essential for the success of any health promotion program or strategy [35]. Furthermore, there is evidence to show that community member skill development is essential to a health promotion program. These strategies are most likely to be successful if combined with other strategies such as providing increased access to goods, products, or services. For example a review of health promotion strategies addressing high-risk behaviours putting youth at risk for HIV/AIDS found that the key to a successful program was providing motivations to change behaviour such as peer education, support, while simultaneously providing products and services needed to achieve the behaviour change. It has been found that the most effective interventions involve a combination of health promotion strategies occurring at the personal, community and structural level [35] Our rigorous peer training, ongoing meetings, and debriefings proved beneficial in building skills in our community peers (unpublished data). Increasing knowledge in peers, increases their self-confidence, enhances empowerment and promotes community capacity building (unpublished data). We are creating permanent mentors, peers and project participants alike, through the process of ongoing knowledge translation. Peer satisfaction, knowledge, skills and self-efficacy will be measured at the end of the project.

Introduction of health interventions to nominated friends of individuals was found to be the best recruitment strategy for the PROMPT study. The most important benefit of this method is scalability, because it can be implemented without mapping the social network [23]. This community-based project utilizes the preexisting and deeply engrained social networks of this community. This strategy has the potential of producing the greatest cascades or spill-over effects and maximal population-level behaviour change. Thus, to encourage the uptake of this tobacco dependence intervention, the PROMPT study has operationalized this social network-based recruitment and retention strategy for maximum efficiency.

The weekly skills workshops created and led by our peers, are useful building blocks for community capacity building and improving self-confidence in peers and project participants. We are currently measuring the impact of and participation in these workshops. Several reviews suggest that creating supportive conditions is essential in order facilitate any health promotion effort. [23] This may include implementing a variety of actions that promote supportive conditions at the structural (policy), social (including community) and individual levels. Therefore, to successfully alter high-risk behaviours, the underlying social and economic conditions must be addressed [35]. Key factors to the success of such interventions are the ability to access support, including the availability of peer counseling, outreach services and skills training.

Empowerment is an action-oriented concept with a focus on removal of formal or informal barriers, and on transforming power relations between communities, institutions and government [34]. Empowerment includes both processes and outcomes, with empowerment of marginalized people being an important outcome in its own right, and also an intermediate outcome in the pathway to reducing health disparities and social exclusion [34]. Therefore, within the context of this “*pathways to health framework”* we have operationalized a comprehensive, ten-step citizen engagement and action model for a tobacco dependence management research program, which involves providing access to smoking cessation aids (access to resources) along with various life-skills training workshops (empowerment) for participants to build community capacity, create structural and social conditions to support the development of personal skills, empower the members involved and to provide support to participants in a low-threshold and safe community setting.

### Challenges of CBPAR research

There are many challenges in operationalizing such community endeavors. Sustainability due to lack of ongoing funding is an ongoing challenge for community-based research [36, 37]. The challenges are partly because the process takes much more time than “traditional” academic research as all community health care partners and community members need to understand and agree on the issues. Building trust and engagement, especially with the most marginalized populations, is very time consuming because historically these populations have been disenfranchised by academicians, policy makers, governments and general population [4]. We have been working on building relationships with community members over several years (one year for The PROMPT study and over three years in the PROUD study). The time factor is important from the academic researchers’ point of view considering implications for academic tenure and promotion. From the community perspective, the implications for the time needed to address the issue under study are significant. This is because, usually community problems are current and solutions are complex and occasionally the concept of a solution could be perceived as farfetched or impossible in their minds. Changing community dynamics due to in- and out-migration and changing academic personnel due to promotions, transfers or retirements are looming threats to the success of community-based action projects. There is a need to reflect on the challenges created by changing personnel when a lengthy relationship between the community and academic partners is required to undertake action research. This is simply because all new and old partners may not have the same needs or outlook [37]. All partners may have different perspectives in a lengthy, evolving relationship. Thus, over time expectations might change and there may not be the capacity to overcome such dynamic challenges in the project. We did encounter the issue of changing academic personnel however, we persevered through the rough transition. Through CBPAR research the goal is to facilitate ongoing recruitment, create partnerships with key community members, anticipate potential difficulties through prior experiences and find strength in collaboration and team building through training and knowledge translation. Our team persevered despite all the above mentioned challenges and still managed to create a supportive environment in which peers and community members could thrive and remain engaged in the project.

### Benefits of CBPAR research

Encouraging community feedback and peer participation throughout every phase of the project has helped the research team better understand the needs of the community. Furthermore, it provided the staff with a better understanding of the services and support required by this specific population in order to address their issues e.g. tobacco dependence. Through this approach, the research staff have gained a deeper understanding of the relationship between smoking and the social determinants of health which affect this community at large. The participatory approach utilized has allowed for knowledge exchanges between both the peer researchers and the academic staff. The academic staff continually gained a better understanding of the inner city community within Ottawa, specifically around communication. The academic ‘jargon’ is a deterrent in the communication process and most often not required for effective communication. Importantly, cultural sensitivity is utmost important in spoken and un-spoken communication; which was learnt by the academic staff by forming closer and collegial relationship with community peers. The peer researchers have gained general knowledge related to skills required for research participation, accountability, time management and specific knowledge related to lung function testing and the impact of smoking on health outcomes. The peers exceeded our expectations about their professionalism, articulateness and their unwavering dedication towards betterment of this most marginalized population. E.g. one of our peer at a community knowledge forum said, ‘*No one is a drug addict or homeless by choice, and no one wants to be there. We need help, not hand-outs!’*

### Future plans

The future plans for the project and for the community research centre are being discussed at our regular peer meetings. The research team and peers have formed a Community Advisory Committee (CAC) comprising of members from our target population and keen key representatives from the neighbourhood healthcare agencies. The CAC is responsible for envisioning future projects and overlooking current projects at the community research centre. There is diversity in the CAC membership, with regards to sexual orientation, indigenous status and francophone representation. PROMPT is currently following 80 participants with two engaged peers. Our partnership with peers and neighborhood organizations is flourishing and has givien rise to a new grassroot organization by our peers to further their mission of harm reduction, advocacy and community capacity building: ONPAHR (Ottawa Network of Peers Acting for Harm Reduction).

## Conclusion

The PROMPT study demonstrated the feasibility of using community-based participatory action research (CBPAR) embedded in social network-based approach to build engagement and trust in the most difficult to reach and hardest to treat inner city populations. The ten-step Ottawa Citizen Engagement and Action Model (OCEAM) was successfully operationalized. The PROMPT study will continue to follow eighty PROMPT participants for six months in order to understand the barriers and facilitators of smoking as well as optimal ways to manage, reduce, and quit smoking. Rather than the heavily ‘acute-care’ or ‘disease-focused’ health research and health policy, focusing on disparity and need of different sub-population groups is urgent [4, 17]. Community-based research emphasizes ecological model of health encompassing physical, mental, biomedical, social, economic, cultural, historical, and political factors as determinants of health and disease [17]. We have successfully demonstrated that the strengths within the inner city community could be harnessed to tackle issues such as tobacco addiction. Through this research, we have made attempts to improve self-confidence and enhance empowerment in peers and participants, and in turn build community capacity. Thus, this strategy is best suited for tackling health inequity and hence, serves the greater purpose of health justice. However, our efforts should always be guided by wisdom, compassion and loving kindness. Importantly, such holistic approaches to chronic diseases such as tobacco dependence are urgently needed [38].

## Ethical approval

The study was approved by the Ottawa Hospital Research Ethics Board; written consent was obtained from all participants.
